# An ORFV F1L mRNA Vaccine Candidate: Preparation, Immunogenicity, and Comparison with a Commercial Live Vaccine

**DOI:** 10.3390/ani16142274

**Published:** 2026-07-22

**Authors:** Yusheng Lin, Jinxiu Jiang, Weiwei Liu, Kul Raj Rai, Yongliang Che

**Affiliations:** 1 Institute of Animal Husbandry & Veterinary Medicine, Fujian Academy of Agricultural Sciences, Fuzhou 350013, China; 2 NAST Biomedical Research Laboratory, Faculty of Science, Nepal Academy of Science and Technology (NAST), GPO Box:3323, Khumaltar, Lalitpur 44700, Nepal

**Keywords:** Orf virus, mRNA vaccine, lipid nanoparticles, immunogenicity, commercial vaccine

## Abstract

Orf virus (ORFV) is a major pathogen in goats and sheep, and control currently depends mainly on commercial live vaccines. Although mRNA vaccines have revolutionized human medicine, their use in veterinary settings is largely unexplored. In this study, an mRNA vaccine candidate encoding the ORFV F1L protein (F1L-mRNA-LNP) was developed via in vitro transcription and encapsulated in lipid nanoparticles. BALB/c mice were divided into five groups (n = 14 each): three receiving different doses of F1L-mRNA-LNP (5, 10, or 15 μg), one receiving a commercial live vaccine (CV), and a PBS control group. Mice were immunized intramuscularly and boosted after 14 days; immune responses were assessed 14 days later following ARRIVE 2.0 guidelines. Both the F1L-mRNA-LNP and CV vaccines induced specific antibodies versus PBS (*p* < 0.01). The 10 μg mRNA group showed Th1 cytokine and CD8^+^ T cell responses comparable to CV (*p* > 0.05), whereas IL-4 (Th2) was significantly higher in the CV group (*p* < 0.05). Neutralizing antibody titers did not differ between groups, indicating that the mRNA vaccine induces comparable Th1 cellular immunity but weaker Th2 humoral immunity. Upon ORFV challenge, the 10 μg F1L-mRNA-LNP vaccine protected BALB/c mice, as evidenced by stable body weight, no clinical symptoms, and reduced viral load, with efficacy comparable to CV (*p* > 0.05). This study provides strong evidence supporting the optimization of ORFV mRNA vaccines and highlights the translational potential of the F1L-mRNA-LNP candidate vaccine for veterinary applications.

## Simple Summary

A new mRNA vaccine candidate encoding the ORFV F1L protein (F1L-mRNA-LNP) was developed and evaluated in mice against a commercial live vaccine. At a 10 μg dose, the mRNA vaccine elicited cellular immune responses comparable to the commercial vaccine and conferred equivalent protective efficacy upon viral challenge. Although humoral antibody responses were relatively lower, the vaccine demonstrated clear protective potential. These findings position F1L-mRNA-LNP as a promising candidate for ORFV control, warranting further preclinical and translational studies.

## 1. Introduction

Orf is an acute, contagious, zoonotic disease caused by Orf virus (ORFV), mainly infecting goats and sheep [1,2]. It is characterized by vesicular, pustular, and crusted lesions on the lips, udder, tail root, and hooves, with a high mortality rate in lambs, causing significant economic losses to the global sheep industry [3,4,5]. The disease is widely distributed worldwide. Since it was first reported in China in 1955, outbreaks have continued to occur in major sheep-raising regions such as Gansu, Shaanxi, Jilin, Inner Mongolia, Shandong, Guangxi, Fujian, and Taiwan, posing severe challenges for disease control [4,6]. ORFV employs a complex immune evasion strategy, encoding multiple immunomodulatory proteins that interfere with both innate and adaptive host immune responses, thereby enabling frequent reinfections even in animals with high antibody titers [1,5,6]. Currently, no specific therapeutic drugs are available, and vaccination remains the primary approach for disease prevention [2,7].

Existing commercial vaccines are primarily live attenuated or inactivated formulations [7]. While they have contributed to disease control, both possess notable limitations: live attenuated vaccines carry a risk of reversion to virulence, and inactivated vaccines are relatively weakly immunogenic, and both types poorly induce cellular immunity, particularly CD8^+^ T cell responses, which are crucial for clearing intracellular viruses [5,6]. Therefore, developing novel vaccines that activate both humoral and cellular immunity is of great scientific and practical significance.

In recent years, mRNA vaccine technology has made substantial advances, exhibiting rapid development timelines, high efficacy, and robust safety profiles in the context of SARS-CoV-2 [8,9,10,11,12,13]. mRNA vaccines are prepared by in vitro transcription, encapsulated in lipid nanoparticles (LNPs), and delivered into cells, where the encoded antigen is translated in the cytoplasm. Antigen presentation via both MHC-I and MHC-II pathways activates CD4^+^ T cells, CD8^+^ T cells, and B cells, eliciting robust humoral and cellular immunity [8,14,15,16,17]. Moreover, mRNA itself possesses intrinsic adjuvant activity, activating innate immunity through pattern recognition receptors such as Toll-like receptors (TLRs), further enhancing immune responses [15,17,18,19,20]. These properties position mRNA vaccines as a highly promising platform for veterinary applications [21,22,23,24,25].

The F1L protein is a major surface structural protein of ORFV, playing a key role in viral attachment and entry, and is highly conserved among different strains, making it an important target antigen for vaccine development [3,6,26]. Previous studies have shown that recombinant protein vaccines [26,27], DNA vaccines [28], and adenovirus-vectored vaccines [6] based on F1L can induce specific antibody and T cell responses and provide partial protection against challenge [3,14,29]. To date, the application of mRNA vaccines against ORFV remains unreported, and no studies have yet directly compared the immune responses elicited by an ORFV mRNA candidate vaccine versus commercial live vaccines.

Based on this background, this study constructed an mRNA vaccine candidate encoding the ORFV F1L protein (F1L-mRNA-LNPs), systematically evaluated its immunogenicity in a mouse model, and performed a parallel comparison with a commercial live vaccine, focusing on the differences in Th1/Th2 immune responses, neutralizing antibodies, and CD8^+^ T cell responses. More importantly, the F1L-mRNA-LNP vaccine protected BALB/c mice upon ORFV challenge. This study provides experimental evidence for the development of ORFV mRNA vaccine candidates and supports the translational potential of F1L-mRNA-LNP candidate vaccine in veterinary application.

## 2. Materials and Methods

### 2.1. Reagents, Bacterial Strains, and Viruses

Trans1-T1 competent cells were purchased from TransGen Biotech (Beijing, China), and the pGEM-3Zf (+) plasmid was obtained from MiaoLing Bio (Wuhan, China). HEK-293T cells and the ORFV FZ strain were maintained in our laboratory. Restriction endonucleases Cla I, Pac I, Kpn I, Xho I, and Hind III were sourced from NEB (Ipswich, MA, USA). The EasyPure^®^ Simple Viral DNA/RNA Kit, 2×TransStart^®^ FastPfu Fly PCR SuperMix, and other related reagents were purchased from TransGen Biotech (Beijing, China). DL 15K DNA Marker and DL2000 DNA Marker were obtained from TaKaRa (Dalian, China), while ColorMixed Protein Marker was sourced from ABclonal (Woburn, MA, USA). The EasyCap T7 Co-transcription Kit with CAG Trimer was purchased from Vazyme (Nanjing, China), and N1-Me-Pseudo UTP was obtained from Aladdin (Shanghai, China). The MEGAclear™ Kit Purification for Large Scale Transcription Reactions was purchased from Thermo Fisher (Waltham, MA, USA). Fetal bovine serum, DMEM medium, trypsin, and the BCA protein assay kit were obtained from Zeta Life (Menlo Park, NJ, USA). The ECL (super) chemiluminescence kit and FuturePAGE™ precast protein gels were purchased from ACE Biotech (Changzhou, China). The Orf virus antibody ELISA kit was sourced from Weikeqi Bio (Sichuan, China), and mouse IFN-γ, IL-2, TNF-α and IL-4 ELISA kits were purchased from Hengyuan Bio (Shanghai, China). PE-conjugated anti-mouse CD4, FITC-conjugated anti-mouse CD8, and PE-Cy5-conjugated anti-mouse CD3ε monoclonal antibodies were obtained from Lianke Bio (Hangzhou, China). The commercial ORFV live vaccine (CV) (HCE strain, veterinary drug approval number: 150104031, lot number: 202401001) was purchased from Shandong Huahong Bioengineering Co., Ltd. (Shandong, China).

### 2.2. Experimental Animals

Six-week-old specific pathogen-free (SPF) female BALB/c mice were purchased from Fuzhou Nodans Biotechnology Co., Ltd. They were housed in an SPF animal facility under controlled conditions: 22 ± 2 °C, 50% ± 10% humidity, and a 12 h light/dark cycle (lights on from 7:00 to 19:00), with free access to standard rodent chow and sterilized water. Mice were maintained in cages measuring 29 × 18 × 13 cm, with corncob bedding changed twice weekly, and no more than five mice per cage.

### 2.3. Anesthesia and Euthanasia

Mice were sacrificed by CO_2_ gassing. All animal experiments were reviewed and approved by the Ethics Committee of the Institute of Animal Husbandry and Veterinary Medicine, Fujian Academy of Agricultural Sciences. Efforts were made to minimize animal suffering.

### 2.4. Primer Design and Synthesis

The sequences of the EGFP gene and the ORFV NZ strain (DQ184476) F1L gene were downloaded from NCBI/GenBank. Specific primers were designed using Oligo7.0 software to amplify the full-length genes. To enhance protein expression, a Kozak sequence (GCCACC) was introduced upstream of the start codon, and a double stop codon was introduced downstream of the original stop codon. The primer sequences are listed in Table S1. All primers were synthesized by Fuzhou Shangya Biotechnology Co., Ltd. (Fuzhou, China).

### 2.5. Construction of the In Vitro Transcription System

The 5′ and 3′ untranslated regions (UTRs) of α-globin were selected, followed by a 120-nt poly(A) tail. The restriction sites Cla I and Pac I were introduced into the multiple cloning site (MCS) for gene insertion, and Kpn I and Hind III were introduced at the ends of the UTRs. The entire sequence was cloned into the pGEM-3Zf (+) vector to construct the in vitro transcription system, named pGEM-UTR.

### 2.6. Construction of Recombinant Plasmids pGEM-EGFP-UTR and pGEM-ORFV-F1L-UTR

The EGFP gene and the ORFV-F1L gene were separately inserted into pGEM-UTR to generate recombinant plasmids pGEM-EGFP-UTR and pGEM-ORFV-F1L-UTR (Figure S1).

### 2.7. Amplification and Purification of Target Genes

The EGFP gene was amplified from the pEGFP plasmid, and the F1L gene was amplified from ORFV vaccine strain DNA using specific primers. The PCR conditions were: 95 °C for 5 min; 35 cycles of 95 °C for 30 s, 55 °C for 30 s, 72 °C for 1 min; and a final extension at 72 °C for 10 min. The PCR products were separated by 1.5% agarose gel electrophoresis, and the target bands were excised under blue light. Gel purification was performed according to the manufacturer’s instructions, yielding fragments named Cla I-EGFP-Pac I-PCR and Cla I-ORFV-F1L-Pac I-PCR.

### 2.8. Restriction Digestion, Ligation, Transformation, and Identification of Recombinant Plasmids

The purified PCR products and pGEM-UTR were double-digested with Cla I and Pac I overnight at 37 °C. The digested products were separated by 1.5% agarose gel electrophoresis, and the target bands were purified, yielding Cla I-EGFP-Pac I, Cla I-ORFV-F1L-Pac I, and Cla I-pGEM-UTR-Pac I. These fragments were ligated and transformed into Trans-T1 competent cells. Colonies were selected on LB (AMP^+^) plates, and plasmids were extracted using a plasmid miniprep kit. Double-digestion with Cla I and Pac I was performed to confirm correct insertion, and positive clones were sequenced by Fuzhou Shangya Biotechnology Co., Ltd. (Fuzhou, China).

### 2.9. Linearization of Recombinant Plasmids

Bacterial clones containing correctly sequenced plasmids were inoculated into 200 mL of LB (AMP^+^) medium and cultured at 37 °C with shaking at 220 rpm for 16 h. The recombinant plasmids pGEM-EGFP-UTR and pGEM-ORFV-F1L-UTR were extracted using an endotoxin-free plasmid maxi-prep kit, and their concentration and purity were measured with a NanoDrop 2000 (Waltham, MA, USA) spectrophotometer.

### 2.10. In Vitro Transcription Incorporating Nucleoside-Modified Nucleotides

In vitro transcription was carried out using the EasyCap T7 Co-transcription Kit supplemented with CAG Trimer. T7 RNA polymerase and the CAG Trimer cap analogue were employed to generate single-stranded RNA bearing a 5′-m7GCap1 structure. In the reaction mixture, uridine triphosphate (UTP) was replaced with N1-methylpseudouridine triphosphate (N1-Me-Pseudo UTP).

### 2.11. Cap-mRNA Purification

Capped mRNA was purified using the MEGAclear™ Kit Purification for Large Scale Transcription Reactions.

### 2.12. In Vitro Cell Transfection of Cap-mRNA

293T cells were transfected with Cap-mRNA using Advanced DNA RNA Transfection Reagent. Sixteen hours after transfection, the medium was removed, cells were washed once with PBS and lysed on ice with RIPA lysis buffer containing 1 mmol·L^−1^ PMSF. After 30 min of lysis, the lysate was centrifuged at 12,000 rpm for 5 min at 4 °C, and the supernatant was collected. Protein concentration was measured using the BCA protein assay kit.

### 2.13. Indirect Immunofluorescence Assay

293T cells transfected with ORFV F1L-encoding mRNA were cultured overnight at 37 °C in a 5% CO_2_ atmosphere. At 24 h post-transfection, cells were fixed with 4% paraformaldehyde for 30 min, washed three times with phosphate-buffered saline (PBS), and permeabilized with 0.3% Triton X-100 for 10 min. Following three additional washes, cells were blocked with 5% bovine serum albumin (BSA) in PBS for 1 h at 37 °C. Subsequently, cells were incubated with rabbit anti-ORFV serum (1:1 dilution) for 1 h at 37 °C. After three washes, cells were incubated with FITC-conjugated HRP-labeled goat anti-rabbit IgG (1:500 dilution) for 1 h at 37 °C, washed three more times, and stained with DAPI for 5 min. Finally, cells were examined using an inverted fluorescence microscope.

### 2.14. Western Blot Assay

293T cells transfected with ORFV F1L-mRNA were harvested after 16 h, and total protein was extracted. Samples were denatured at 100 °C for 5 min and separated by SDS-PAGE. Proteins were transferred onto PVDF membranes, blocked with 5% non-fat milk for 2 h at room temperature, and incubated overnight at 4 °C with rabbit anti-ORFV polyclonal antibody. After three washes with TBST, membranes were incubated with HRP-conjugated goat anti-rabbit IgG for 1 h at room temperature, washed three times, and visualized using an imaging system.

### 2.15. Lipid Nanoparticle Preparation, Electron Microscopy, and Particle Size and Zeta Potential Measurement

Lipid nanoparticles (LNPs) encapsulating mRNA (F1L-mRNA-LNPs) were prepared at an N/P ratio of 8:1 by Nanjing Genscript Biotechnology Co., Ltd. (Nanjing, China). (https://www.genscript.com.cn/accessed on 8 March 2024) to generate the mRNA vaccine candidate. Briefly, LNPs were formulated using a microfluidic mixing system (Microfluidic Mixing System-2). The ionizable lipid (SM102), DSPC, cholesterol, and DMG-PEG2000 were dissolved in ethanol at predetermined optimal molar ratios. mRNA was dissolved in citrate buffer (pH 4.0). The lipid and mRNA solutions were rapidly mixed at a controlled flow rate ratio using a microfluidic device, facilitating LNP self-assembly through spontaneous phase mixing. The resulting LNPs were dialyzed against phosphate-buffered saline (PBS, pH 7.4) to remove ethanol and adjust the pH. LNPs were appropriately diluted and visualized using transmission electron microscopy (TEM). Particle size and zeta potential were measured using a Malvern Zetasizer Nano ZS (Malvern, UK). All measurements were performed in triplicate. The LNP formulation process included sterile filtration (0.22 μm) to ensure microbial safety.

### 2.16. Mouse Immunization and Immunological Assays

Six-week-old SPF female BALB/c mice were randomly divided into five groups (n = 14 per group). Mice in the F1L-mRNA-LNP groups received intramuscular injections of 50 μL of the mRNA-LNP formulation at doses of 5 μg, 10 μg, or 15 μg per mouse, respectively. The commercial live vaccine (ORFV HCE strain; Shandong Huahong Bioengineering Co., Ltd. (Shandong, China); veterinary drug approval number: 150104031) was diluted in sterile phosphate-buffered saline (PBS) to a final volume of 50 μL per mouse prior to administration. Mice in the control group received 50 μL of sterile PBS alone. Mice were immunized intramuscularly in the hind leg and boosted 14 days later using the same dose and route. Sample size (n = 14 per group) was chosen based on preliminary experimental data and the need to have sufficient mice for both serological analysis and terminal splenocyte collection at multiple time points, ensuring adequate statistical power to detect a meaningful difference in antibody titers and T cell responses as described previously [30,31].

### 2.17. Sample Collection and Antibody Detection

Blood samples were collected from the tails of the mice at various time points: before the immunization (on day 0), on day 14 (before the booster immunization), on day 21, on day 28, on day 35, and on day 42. Serum was separated and stored at −20 °C. ORFV-specific antibodies were measured by ELISA using the Orf virus antibody ELISA kit (Weikeqi Bio, Chengdu, China). Neutralizing antibody titers (NT50) were determined by microneutralization assay: sera were heat-inactivated at 56 °C for 30 min, serially diluted (1:4 to 1:512), mixed with 100 TCID50 of ORFV FZ strain, incubated for 1 h at 37 °C, and then added to BHK-21 cell monolayers. After 72 h, cytopathic effect (CPE) was recorded, and the NT50 was defined as the highest dilution that inhibited 50% of CPE.

### 2.18. T Lymphocyte Subset Analysis

At day 28, three mice from each group were euthanized by cervical dislocation under deep anesthesia as described above, and spleens were collected. Splenocytes were isolated by mechanical disruption and passed through 70 μm cell strainers. Red blood cells were lysed with ACK lysis buffer. Cells were washed and resuspended in PBS with 2% FBS. For T cell subset analysis, 1 × 10^6^ cells were stained with anti-mouse CD3ε-PE-Cy5 (clone 145-2C11, 1:200), CD4-FITC (clone GK1.5, 1:200), and CD8-PE (clone 53-6.7, 1:200) for 30 min at 4 °C. Samples were acquired on a CytoFLEX flow cytometer (Beckman Coulter, Indianapolis, IN, USA), and data were analyzed using FlowJo v10. The gating strategy was as follows: lymphocytes were gated by FSC/SSC, followed by CD3^+^ selection, and then CD4^+^ and CD8^+^ subpopulations.

### 2.19. Cytokine Detection

Splenocytes (2 × 10^6^/mL) were stimulated with recombinant F1L protein (10 μg/mL) in 24-well plates for 48 h at 37 °C with 5% CO_2_. Supernatants were collected, and cytokines (IFN-γ, IL-2, TNF-α, IL-4) were measured using commercial ELISA kits (Hengyuan Bio) according to the manufacturer’s protocols. All samples were assayed in duplicate.

### 2.20. Viral Challenge and Protection Assessment

The ORFV FZ strain used for challenge was isolated and propagated in BHK-21 cells, and viral titers were determined as TCID_50_ prior to inoculation. At 14 days post-boost (day 28), mice (n = 6 per group) were challenged intramuscularly in the contralateral hind leg with 10^5^ TCID_50_ of ORFV FZ strain in 50 μL PBS. Mice were monitored daily for 7 days post-challenge for clinical signs, body weight, and lesion development. Lesion severity was scored on a 0–4 scale as follows: 0, no visible lesion; 1, mild erythema at injection site; 2, moderate swelling with localized erythema; 3, severe swelling with crust formation; 4, ulceration or scab formation extending beyond injection site. Viral load assessment: Tissues were collected at 7 days post-challenge. Viral DNA was extracted using the EasyPure^®^ Simple Viral DNA/RNA Kit, and ORFV F1L gene copies were quantified by qPCR using SYBR Green detection. Standard curves were generated using serial dilutions of ORFV F1L plasmid DNA. All qPCR reactions were performed in triplicate.

### 2.21. Statistical Analysis

Data were analyzed using GraphPad Prism 8.0. All data are expressed as mean ± SD. Normality was assessed using Shapiro–Wilk test; all data passed normality. Homogeneity of variance was confirmed by Levene’s test. Comparisons among multiple groups were performed using one-way ANOVA followed by Dunnett‘s post hoc test for comparisons against the PBS control, or Tukey’s test for pairwise comparisons between all groups. Comparisons between two groups were performed using a two-tailed Student’s *t* test. A *p* value of less than 0.05 was considered statistically significant. Exact *p* values are reported where applicable (e.g., *p* = 0.023). In figures and tables, significance is denoted as **** *p* < 0.0001,*** *p* < 0.001,** *p* < 0.01, * *p* < 0.05, ns (*p* > 0.05).

## 3. Results

### 3.1. Amplification of Target Genes and Construction of Recombinant Plasmids

**Figure 1:**
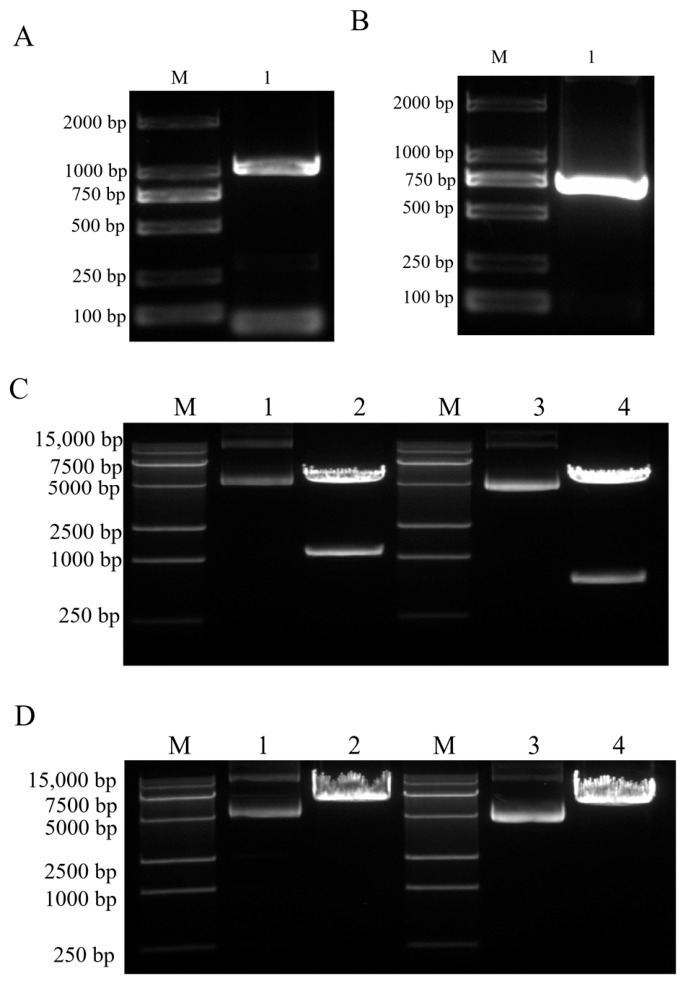
Amplification of target genes and construction of recombinant plasmids Agarose gel electrophoresis results showing (**A**) PCR product of ORFV-F1L gene (~1011 bp); (**B**) PCR product of EGFP gene (~720 bp). (**C**) Double restriction enzyme digestion of recombinant plasmids pGEM-ORFV-F1L-UTR and pGEM-EGFP-UTR with ClaI and PacI. Lane M: DL15K DNA marker; lane 1: uncut pGEM-ORFV-F1L-UTR; lane 2: pGEM-ORFV-F1L-UTR digested with ClaI and PacI; lane 3: uncut pGEM-EGFP-UTR; lane 4: pGEM-EGFP-UTR digested with ClaI and PacI. (**D**) Linearization of recombinant plasmids by XhoI single restriction enzyme digestion. Lane M: DL15K DNA marker; lane 1: uncut pGEM-ORFV-F1L-UTR; lane 2: pGEM-ORFV-F1L-UTR linearized by XhoI; lane 3: uncut pGEM-EGFP-UTR; lane 4: pGEM-EGFP-UTR linearized by XhoI. Shown are representative data from three biologically independent experiments (n = 3).

The target genes, EGFP (720 bp) and ORFV-F1L (1011 bp), were successfully amplified via PCR. Agarose gel electrophoresis (1.5%, *w*/*v*) showed amplicons of the expected sizes for both genes (Figure 1A,B). Subsequent sequencing confirmed the identity and correctness of the amplified sequences. Recombinant plasmids pGEM-EGFP-UTR and pGEM-ORFV-F1L-UTR were double-digested with the restriction enzymes ClaI and PacI. The digestion products were resolved by 1.5% agarose gel electrophoresis and exhibited bands of the expected sizes (Figure 1C), indicating the presence of the target inserts within the recombinant plasmids. Correctly identified recombinant plasmids were linearized by single-digestion with Xho I. The linearized products exhibited the expected band sizes on 1.5% agarose gel electrophoresis (Figure 1D).

### 3.2. In Vitro Expression of EGFP and ORFV F1L Protein

**Figure 2:**
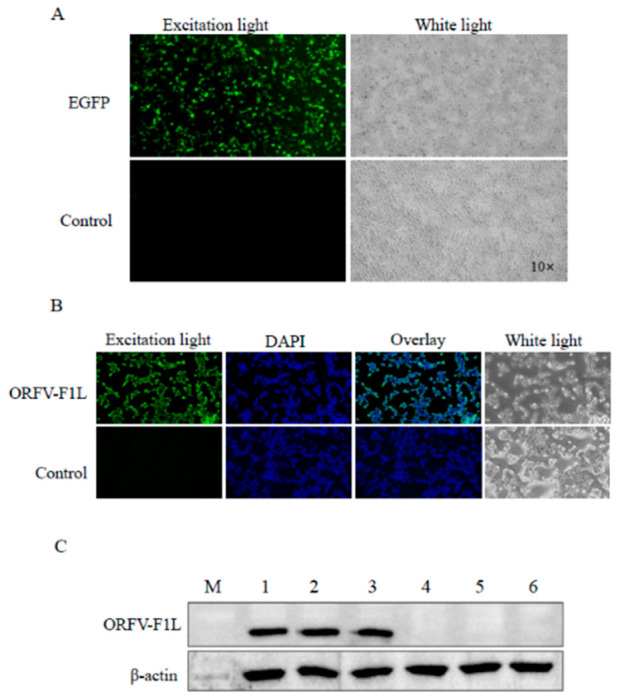
Expression of EGFP and ORFV F1L protein in transfected 293T cells. (**A**) Microscopic examination of cells transfected with Cap-EGFP-UTR (4 μg) or untransfected as control after sixteen hours after transfection under an inverted fluorescence microscope (**B**) Microscopic examination of cells were transfected with vector Cap-mORFV-F1L-UTR (4 μg) or untransfected as control. After, twenty-four hours post-transfection, cells were fixed and stained with rabbit anti-ORFV serum (1:100) followed by FITC-conjugated goat anti-rabbit IgG (green). Nuclei were counterstained with DAPI (blue). (**C**) 293T cells were transfected with Cap-mORFV-F1L-UTR (4 μg) or left untransfected as a control. Twenty-four hours of post-transfection, cells were harvested and lysed for total protein extraction. Protein lysates were subjected to Western blotting using a rabbit anti-ORFV polyclonal antibody. Lane M: ColorMixed protein marker; lanes 1–3: cells transfected with Cap-mORFV-F1L-UTR; lanes 4–6: untransfected control cells. Shown are representative data from three biologically independent experiments (n = 3).

Using florescence microscopy, EGFP fluorescence was observed in 293T cells transfected with Cap EGFP UTR, whereas no fluorescence was detected in control cells, indicating successful expression of the fluorescent reporter protein (Figure 2A). Indirect immunofluorescence assays and Western blotting confirmed expression of the ORFV F1L protein in 293T cells transfected with Cap mORFV F1L UTR (Figure 2B,C).

### 3.3. Physicochemical Characterization of F1L-mRNA-LNP and Preliminary Safety Evaluation

**Figure 3:**
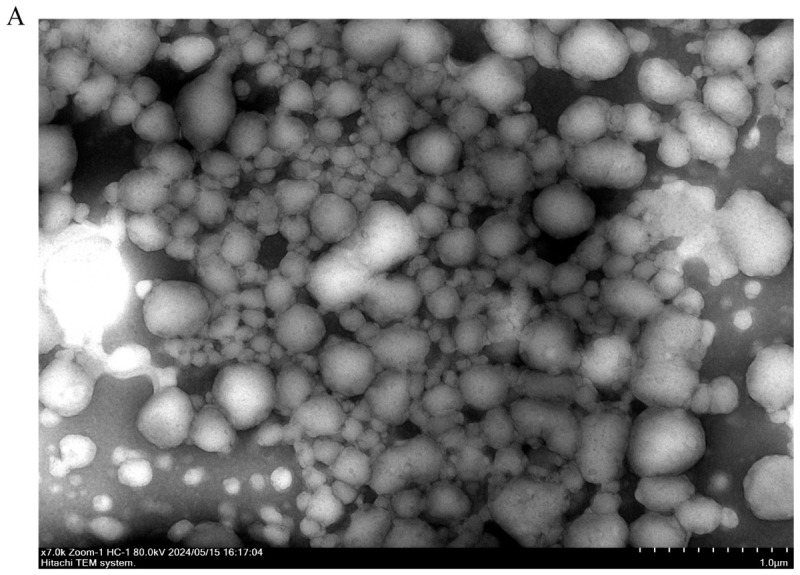

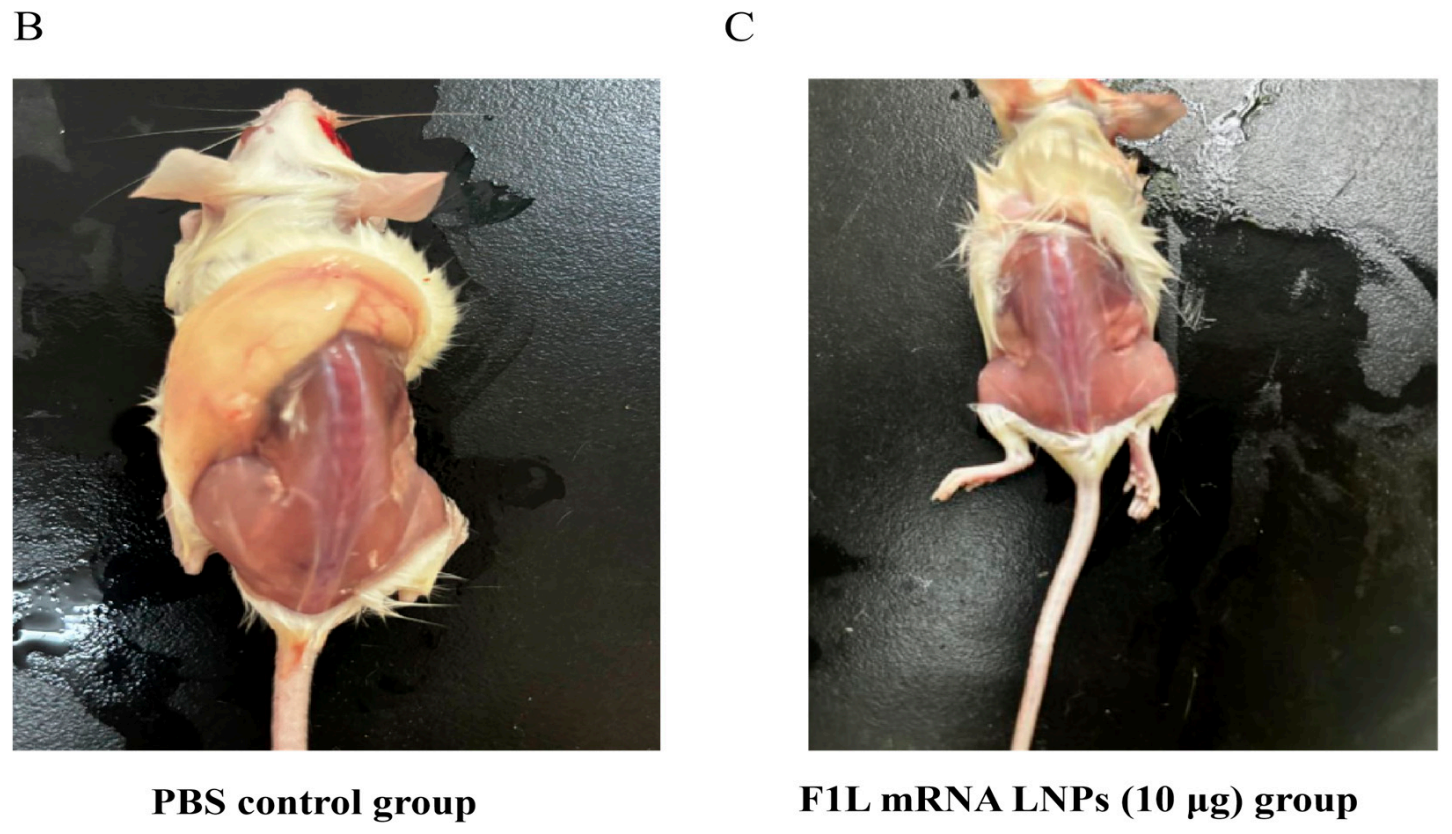
Physicochemical characterization of F1L-mRNA-LNP and preliminary safety evaluation. (**A**) LNPs were prepared at an N/P ratio of 8:1 and observed under a transmission electron microscope. Scale bar = 1 μm. LNPs exhibited spherical morphology with uniform size distribution. Shown are representative data from three biologically independent experiments. (**B**) PBS control group (n = 11). (**C**) F1L mRNA LNPs (10 μg) group (n = 11). Mice were immunized via intramuscular injection in the hind leg with the indicated formulations. Fourteen days after the booster immunization, mice were euthanized, and the muscle tissue at the injection site was exposed and photographed. No visible local reactions (e.g., redness, swelling, or ulceration) were observed in either group.

F1L-mRNA-LNPs (lipid nanoparticles encapsulating messenger RNA encoding the F1L protein) were prepared by Nanjing Genscript Biotechnology Co., Ltd., and the physicochemical properties of the F1L-mRNA-LNPs were characterized. Dynamic light scattering analysis revealed that the nanoparticles exhibited an average hydrodynamic diameter of approximately 100 nm with a polydispersity index (PDI) of 0.135, indicating a relatively homogeneous size distribution. The encapsulation efficiency of mRNA within the LNPs was determined to be 90.8% (Figure S2). Morphological assessment by transmission electron microscopy (TEM) confirmed that the LNPs were spherical in shape with a uniform appearance (Figure 3A). Following administration, no mortality or obvious local or systemic adverse reactions were observed in any LNP-treated group throughout the study period. Daily monitoring revealed no injection site reactions (redness, swelling, or ulceration) at any dose level. All vaccinated mice maintained normal activity, grooming behavior, and feeding patterns comparable to PBS controls (Figure 3B), suggesting a favorable safety profile for the formulated vaccine candidate.

### 3.4. Kinetics of Specific Antibody Responses, Neutralizing Antibody Titers, T Lymphocyte Subset Analysis, Cytokine Levels (ELISA)

**Figure 4:**
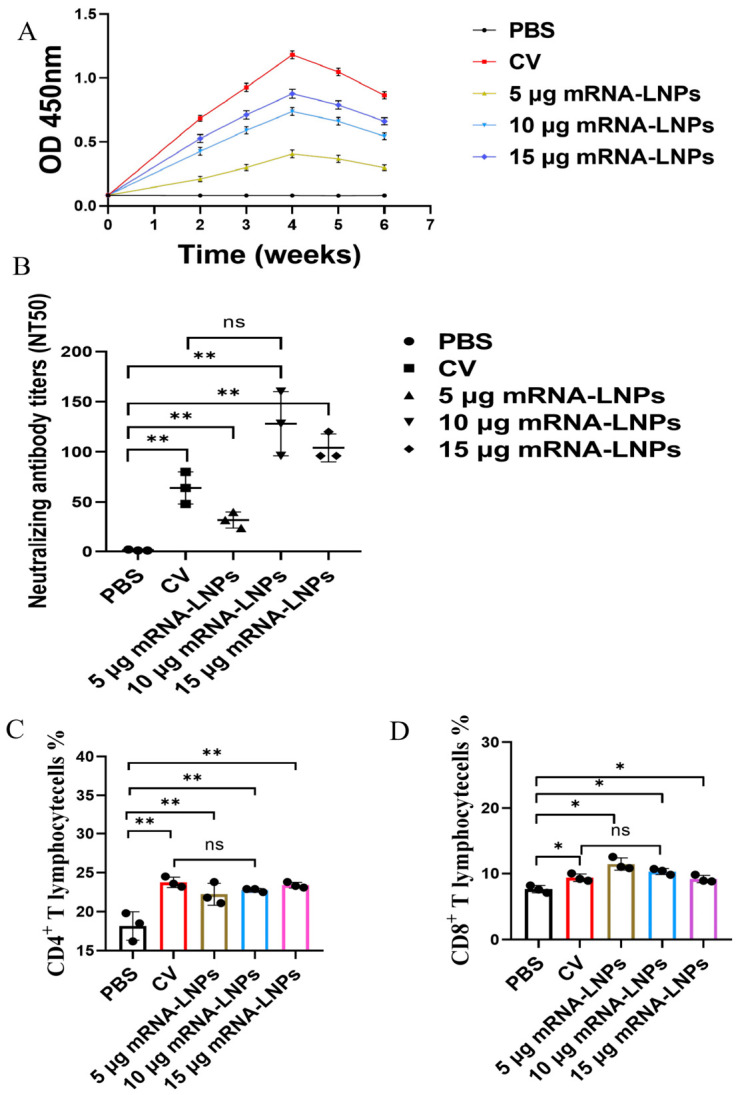
ORFV-specific antibody responses and CD4^+^ and CD8^+^ T cell responses. (**A**) Kinetics of ORFV-specific antibody responses measured by ELISA after intramuscular immunization with F1L mRNA LNPs (5, 10, 15 μg), commercial live vaccine (CV), or PBS control on days 0 and 14. (**B**) Neutralizing antibody titers (NT50) in sera collected on day 28, determined by microneutralization assay (ORFV FZ strain, 100 TCID50; BHK-21 cells). Data are mean ± SD with individual points (n = 10 per group). * *p* < 0.05, ** *p* < 0.01 vs. PBS; ns, not significant (*p* > 0.05); one-way ANOVA with Dunnett’s posttest. (**C**,**D**) Frequency of CD4^+^ (**C**) and CD8^+^. (**D**) T cells following the same immunization regimen as in (**A**,**B**). Statistical comparisons as in (**B**).

Specific antibodies were detected in all F1L-mRNA-LNP dose groups and the CV group at 14 days after primary immunization (before the boost), whereas the PBS group remained negative. At 14 days after the boost, antibody levels in all immunized groups were significantly higher than those in the PBS group (*p* < 0.01) (Figure 4A). Total antibody levels did not differ significantly between the two platforms. Neutralizing antibody titers (NT50) were as follows: F1L-mRNA-LNP (10 μg), 128 ± 32; (15 μg), 96 ± 24; (5 μg), 32 ± 8; CV, 64 ± 16; PBS, <4 (Figure 4B). No significant difference was observed between the F1L-mRNA-LNP (10 μg) and CV groups (*p* > 0.05).

Flow cytometry analysis at 14 days after the boost revealed that CD3^+^CD4^+^ and CD3^+^CD8^+^ T cell percentages were significantly higher in the F1L-mRNA-LNP and CV groups than in the PBS group. No significant differences were observed in CD4^+^ or CD8^+^ T cell percentages between the CV and F1L-mRNA-LNP groups (*p* > 0.05) (Figure 4C,D), indicating comparable T cell differentiation capacity.

Cytokine levels in splenocyte supernatants following F1L protein stimulation are presented in Table 1. No significant differences in Th1 cytokine (IFN-γ, IL-2, TNF-α) levels were observed between the F1L-mRNA-LNP (10 μg) group and the CV group (*p* > 0.05), indicating comparable Th1 cellular immune responses. Th2 cytokine (IL-4, IL-6) levels were significantly higher in the CV group than in the 10 μg and 15 μg mRNA vaccine groups (*p* < 0.05), suggesting that the commercial live vaccine induced stronger Th2 humoral immunity.

### 3.5. Summary of Immune Response Characteristics

Key immune parameters for the two vaccine platforms are summarized in Table 2. Th1 cytokine levels and CD8^+^ T cell percentages did not differ significantly between the F1L-mRNA-LNP (10 μg) and CV groups, whereas Th2 cytokine (IL-4) levels were significantly higher in the CV group.

### 3.6. In Vivo Protective Efficacy Against ORFV Challenge

**Figure 5:**
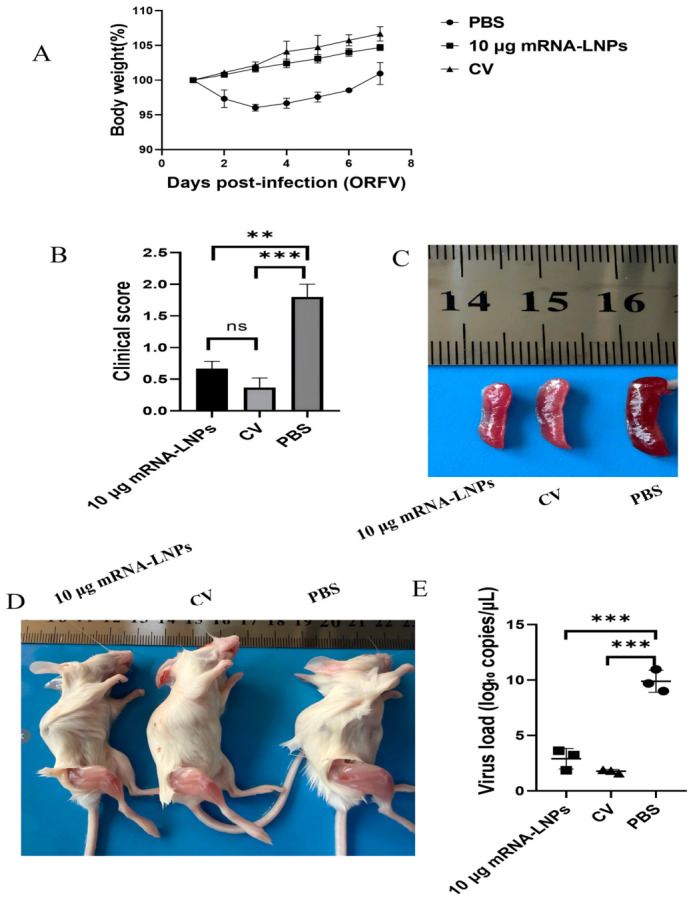
In vivo protective efficacy against ORFV challenge. (**A**) Body weight changes over time, expressed as the percentage of pre-challenge body weight. Mice in the PBS control group showed a significant weight loss from day 2 post-infection, whereas both the F1L-mRNA-LNP (10 μg) and commercial live vaccine (CV) groups maintained stable weights with no significant difference (*p* > 0.05). (**B**) Clinical scores recorded post-challenge. Clinical scores were comparable between the F1L-mRNA-LNP (10 μg) and CV groups (*p* > 0.05). (**C**,**D**) Representative photographs of spleen and lesion sites taken at day 7 post-challenge for each group. (**E**) Viral loads in lesion tissues measured at day 7 post-challenge by qPCR targeting the ORFV F1L gene. Both the F1L-mRNA-LNP (10 μg) and CV groups exhibited significantly lower viral loads than the PBS group, with no significant difference between the two vaccinated groups at any time point (*p* > 0.05). Data are presented as mean ± SD (n = 6 mice per group). Statistical comparisons were performed using one-way ANOVA with appropriate post hoc tests. ** *p* < 0.01, *** *p* < 0.001 compared with PBS group; ns, not significant (*p* > 0.05).

To evaluate the protective efficacy of the F1L-mRNA-LNP vaccine, BALB/c mice were challenged with ORFV FZ strain. Body weight changes were monitored as an indicator of disease severity. Mice in the PBS control group showed a significant weight loss from day 2 post-infection, with recovery beginning by day 4. In contrast, both the F1L-mRNA-LNP (10 μg) and commercial live vaccine (CV) groups maintained stable body weights, with no significant difference from pre-challenge levels (*p* > 0.05) (Figure 5A).

The PBS group developed mild clinical signs, limited to slight redness and swelling at the injection site. No clinical symptoms were observed in either the F1L-mRNA-LNP (10 μg) or CV groups throughout the observation period, and clinical scores were comparable between the two vaccinated groups (*p* > 0.05) (Figure 5B–D).

Viral loads, quantified by qPCR, were significantly lower in both the F1L-mRNA-LNP (10 μg) and CV groups compared to the PBS group (Figure 5E). No significant differences in viral load were detected between the vaccine and CV groups at any time point (*p* > 0.05).

## 4. Discussion

### 4.1. Platform Construction and In Vitro Validation

In the present study, an mRNA vaccine candidate encoding the ORFV F1L protein was successfully constructed and evaluated for immunogenicity in a BALB/c mouse model. To our knowledge, this is the first study on ORFV F1L mRNA vaccine candidate preparation and systematic comparison of the mRNA vaccine candidate with a commercially available live ORFV vaccine. The findings demonstrate that both platforms elicit robust antigen-specific immune responses; however, the nature of the immune profiles differs substantially.

### 4.2. Differential Immune Profiles Between mRNA and Commercial Live Vaccines

The mRNA vaccine induced Th1-biased cellular immunity, characterized by significant production of IFN-γ, IL-2, and TNF-α, along with robust CD8^+^ T cell responses, comparable to those observed in the commercial live vaccine group. In contrast, Th2-associated humoral immunity, characterized by IL-4 levels, was markedly weaker in mRNA-immunized mice. This differential immune bias is consistent with the known intrinsic properties of mRNA-based platforms, which preferentially stimulate Th1-type responses through activation of pattern recognition receptors such as TLR3, TLR7, and TLR8 [15,17,20]. The commercial live vaccine, conversely, contains adjuvants that favor Th2-polarized immunity, which aligns with the stronger Th2 cytokine responses observed in the present study.

### 4.3. Neutralizing Antibody Responses and Protective Potential

Notably, neutralizing antibody titers did not differ significantly between the mRNA vaccine (10 μg dose) and the commercial vaccine groups, suggesting that the mRNA platform is capable of inducing functionally relevant humoral immunity despite eliciting a comparatively weaker Th2 response. This finding underscores the potential of mRNA vaccines to achieve protective antibody responses through alternative pathways, including direct activation of follicular helper T cells and germinal center B cell responses [8,13,18].

### 4.4. CD8^+^ T Cell Responses and Implications for ORFV Control

The comparable CD8^+^ T cell responses between the two platforms are particularly noteworthy, as CD8^+^ T cells play a critical role in controlling ORFV infection by eliminating virus-infected cells [5,19]. The ability of the mRNA vaccine to elicit strong CD8^+^ T cell responses is likely attributable to the endogenous expression of the F1L antigen following translation of the mRNA, which facilitates MHC class I presentation, a key advantage over conventional inactivated or subunit vaccines [13,23,31,32].

Indeed, the present study revealed that the F1L-mRNA-LNP candidate induced Th1-biased cellular immunity and neutralizing antibody responses comparable to those of the commercial live vaccine, while eliciting a relatively weaker Th2 humoral immune response. This difference may be attributed to fundamental variations in how each vaccine platform engages the host immune system. The commercial live vaccine, being replication-competent in vivo, likely provides prolonged antigen exposure and additional danger-associated molecular patterns that favor Th2 pathway activation [26]. Of note, the combination of strong Th1 polarization, robust antibody production, and reduced Th2 responses has been recognized as a characteristic feature of mRNA-LNP platforms, contributing to their effectiveness against infectious diseases, as demonstrated for COVID-19 vaccines [25,31].

### 4.5. Protective Efficacy of the Candidate Vaccine

Consistent with the observed immunogenicity, the F1L-mRNA-LNP vaccine conferred protection against live ORFV challenge in BALB/c mice. Vaccinated animals maintained stable body weights and remained free of clinical symptoms, whereas PBS-treated controls exhibited significant weight loss and mild local lesions. These findings directly demonstrate that the mRNA vaccine-induced immune responses could be translated into protective efficacy. Notably, the protective effects of the F1L-mRNA-LNP vaccine were comparable to those of the commercial live vaccine, as evidenced by similar body weight trajectories, clinical scores, and absence of visible lesions between the two groups. Furthermore, viral genes in lesion tissues were significantly reduced in both vaccinated groups relative to controls, with no statistical difference between the mRNA and commercial vaccine groups. These viral loads reduction is likely attributable to the robust CD8^+^ T cell responses and neutralizing antibodies elicited by the mRNA platform, which together limit viral replication and spread.

### 4.6. Dose–Response Evaluation and Dose Selection for Challenge Experiment

Our dose–response evaluation revealed distinct protective profiles among the three F1L-mRNA-LNP doses. The 5 μg dose elicited suboptimal immune responses, potentially translating into incomplete protection upon ORFV challenge. Conversely, the 15 μg doses did not confer significantly enhanced protection compared to the 10 μg group, indicating a plateau effect where increasing mRNA dose beyond a certain threshold may fail to proportionally improve efficacy, a pattern consistent with previous mRNA vaccine studies [33,34]. Notably, the 10 μg dose achieved protection comparable to the commercial live vaccine, maintaining stable body weight, preventing clinical symptoms, and significantly reducing viral loads. These findings support the selection of 10 μg may be the optimal dose for this vaccine candidate in our experimental model.

While this study successfully developed an ORFV mRNA vaccine candidate and characterized its immunogenicity, some limitations should be acknowledged that may inform the interpretation of the findings and guide future research. For example, immune responses were assessed only at a short-term time point (14 days post-boost), leaving the duration of vaccine-induced immunity uncharacterized. Flow cytometric evaluation was conducted at a single post-boost time point (day 28) to assess peak memory T cell responses; however, serial sampling would better characterize lymphocyte activation kinetics and long-term memory establishment. Additionally, while CD4^+^ and CD8^+^ populations were analyzed, the absence of memory markers such as CD44 and CD62L precludes finer discrimination between central memory (TCM) and effector memory (TEM) subsets, thereby limiting a direct evaluation of long-term immunological memory. Furthermore, the mRNA vaccine elicited a comparatively weaker Th2 humoral immune response relative to the commercial live vaccine. More importantly, the intradermal inoculation mouse challenge model described by Cargnelutti et al. (2011) [35] would yield more robust and reliable findings than the challenge model employed in the present study. Future efforts focusing on sequence optimization, refinement of the lipid nanoparticle formulation, or the inclusion of additional adjuvants may further enhance the immunogenicity of this mRNA vaccine candidate. Moreover, comprehensive stability studies, particularly thermal stability assessments, are important to consider, given that mRNA-LNP vaccines generally demand rigorous cold-chain maintenance; therefore, evaluating the long-term stability of the F1L-mRNA-LNP formulation across a range of temperature conditions will be critical for its viable deployment in veterinary field settings. Future work will also incorporate multiple time points and an expanded marker panel to more fully define the duration and quality of immunity induced by the F1L-mRNA-LNP vaccine. Addressing these limitations will be important for advancing this candidate toward potential veterinary applications.

## 5. Conclusions

This study demonstrates that an F1L-encoding mRNA-LNP vaccine candidate elicits Th1-biased and CD8^+^ T cell responses comparable to those induced by a commercial live ORFV vaccine, with comparable neutralizing antibody titers. Importantly, the vaccine candidate provided significant protective efficacy against live ORFV challenge. Thus, these findings support the F1L-mRNA-LNP vaccine as a promising candidate for further optimization and potential application in veterinary medicine.

## Figures and Tables

**Table 1:** Cytokine levels in mouse splenocytes (pg/mL).

Cytokine	5 μg mRNA-LNPs	10 μg mRNA-LNPs	15 μg mRNA-LNPs	CV	PBS	*p* Value (5 μg vs. CV)	*p* Value (10 μg vs. CV)	*p* Value (15 μg vs. CV)
IFN-γ	182.3 ± 28.6	286.5 ± 42.3	265.2 ± 38.4	156.2 ± 31.5	10.8 ± 2.8	>0.05	>0.05	>0.05
IL-2	98.5 ± 16.2	152.3 ± 25.6	138.6 ± 22.5	86.5 ± 12.3	7.5 ± 1.9	>0.05	>0.05	>0.05
TNF-α	62.3 ± 10.5	98.5 ± 15.2	88.4 ± 13.6	52.3 ± 8.6	5.2 ± 1.3	>0.05	>0.05	>0.05
IL-4	215.6 ± 35.2	198.2 ± 32.1	185.3 ± 30.8	212.4 ± 38.6	9.8 ± 2.1	>0.05	<0.05	<0.05

**Table 2:** Comparison of immune responses induced by F1L-mRNA-LNP and commercial ORFV live vaccine.

Parameter	10 μg mRNA-LNPs	CV	*p* Value
Total antibody titer (OD450)	1.85 ± 0.21	1.72 ± 0.18	>0.05
Neutralizing antibody titer (NT50)	128 ± 32	64 ± 16	>0.05
IFN-γ (ELISA, pg/mL)	286.5 ± 42.3	156.2 ± 31.5	>0.05
IL-2 (ELISA, pg/mL)	152.3 ± 25.6	86.5 ± 12.3	>0.05
TNF-α (ELISA, pg/mL)	98.5 ± 15.2	52.3 ± 8.6	>0.05
IL-4 (ELISA, pg/mL)	198.2 ± 32.1	212.4 ± 38.6	<0.05
CD4^+^ T cells (%)	22.7 ± 1.2	23.7 ± 1.4	>0.05
CD8^+^ T cells (%)	10.6 ± 2.1	9.3 ± 1.5	>0.05

## Data Availability

All the data generated or analyzed in this study are available in this manuscript and supplementary information files. This study did not generate new unique reagents. The F1L-mRNA-LNP vaccine described in this study is available from the corresponding author upon reasonable request. Requests for further information and resources should be directed to and will be fulfilled by the lead contact, Yusheng Lin (15080455134@163.com).

## Supplementary Materials

The following supporting information can be downloaded at: https://www.mdpi.com/article/10.3390/ani16142274/s1, Table S1. PCR primer sequences; Figure S1. Schematic diagram of the in vitro transcription plasmid pGEM-ORFVF1L-UTR; Figure S2. Certificate of analysis for the F1L-mRNA-LNPs (LNP size distribution and Zeta potential); S. Western blot and PCR raw images.
