# Supplementary material for: An ORFV F1L mRNA Vaccine Candidate: Preparation, Immunogenicity, and Comparison with a Commercial Live Vaccine
Source: Animals (Basel). 2026 Jul 22;16(14):2274. doi: 10.3390/ani16142274 (PMC13405235; doi:10.3390/ani16142274)
Supplement: Supplementary file 1 [file animals-16-02274-s001.zip › animals-4421709-Figures S1&S2.pdf]

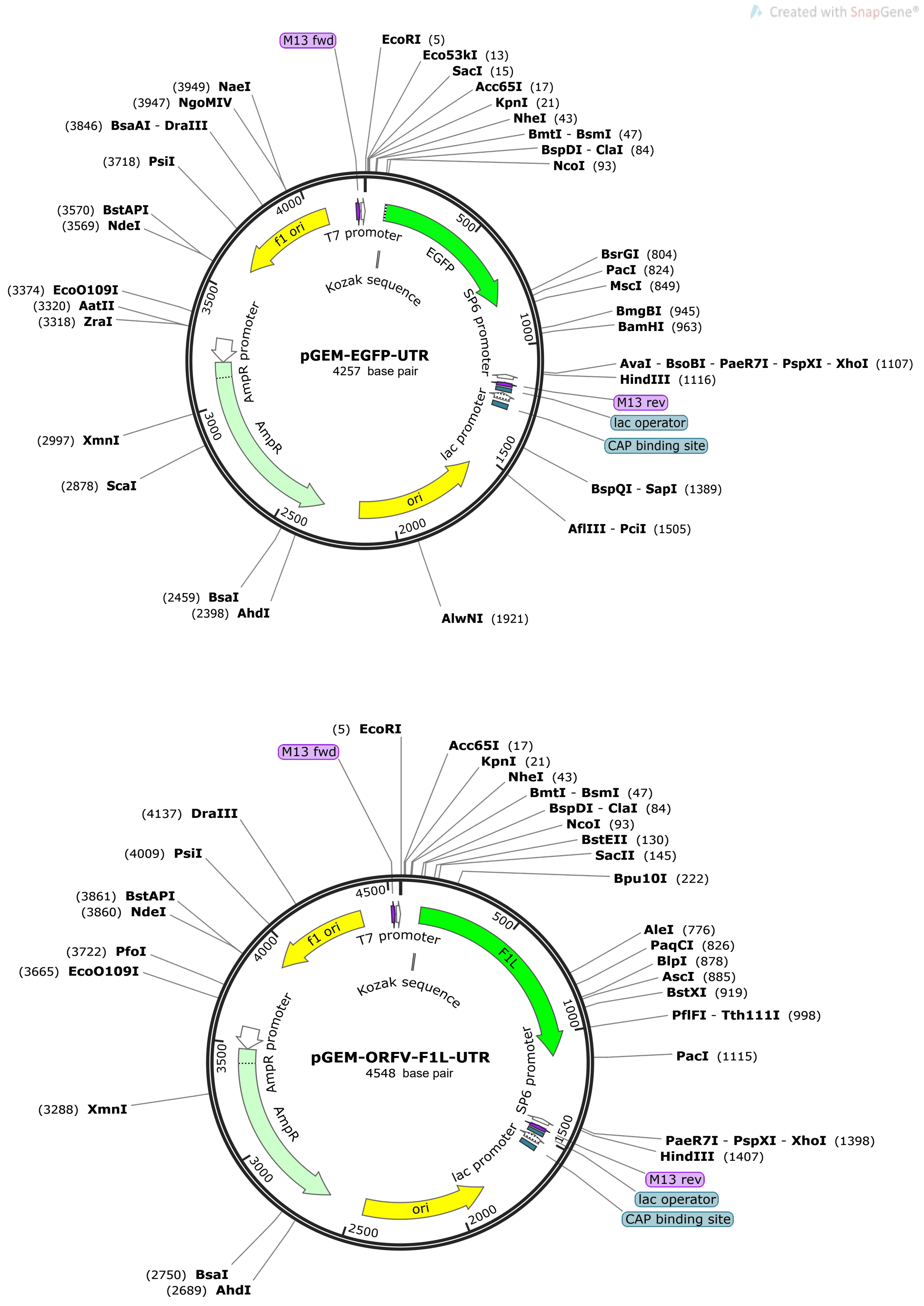

Figuer S1: Schematic diagram of the in vitro transcription plasmid pGEM-ORFV-F1L-UTR.

QC Items	Test methods	Specification	Results
Appearance	Visual Inspect	Clear and free of foreign particles	Pass
pH	pH paper	7.4 ± 0.5	Pass
Final payload concentration	Ribogreen assay	0.1 ~ 0.4 mg/mL	0.35 mg/mL, 0.3 mg/vial, 1 vial
Encapsulation Efficiency	Ribogreen assay	> 85 %	90.80 %
Z-average Size	Dynamic Light Scattering	POI ± 20 nm, 65 - 125 nm	104.8 nm
Polydispersity Index (PDI)	Dynamic Light Scattering	< 0.2	0.1354
Zeta potential	Laser Doppler electrophoresis (LDE)	± 15.0 mV	-5.848mV
Endotoxin	LAL Semi-quantitative	< 4 EU/mL	Pass

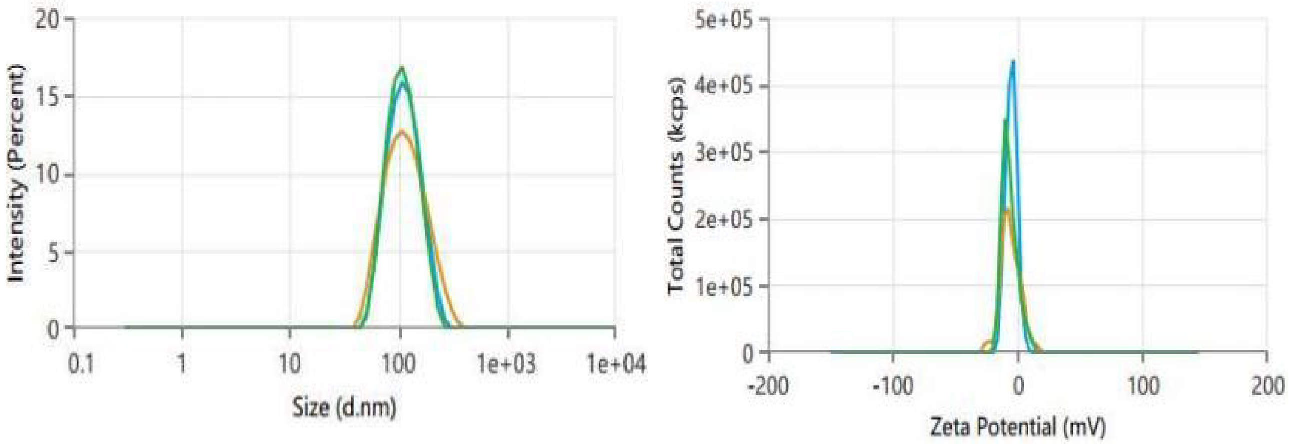

A: Size Distribution by intensity B: Zeta potential Distribution

Figure S2: Certificate of analysis for the F1L-mRNA-LNPs
