# Supplementary material for: An ORFV F1L mRNA Vaccine Candidate: Preparation, Immunogenicity, and Comparison with a Commercial Live Vaccine
Source: Animals (Basel). 2026 Jul 22;16(14):2274. doi: 10.3390/ani16142274 (PMC13405235; doi:10.3390/ani16142274)
Supplement: Supplementary file 1 [file animals-16-02274-s001.zip › animals-4421709-Table S1 PCR primer sequences.pdf]

Table S1 PCR primer sequences

Primer name	Sequence (5′-3′)	Restriction site	Product size (bp)
EGFP-F	GCATCGATGCCACCATGGAAGATGCCAAAAACA	*Cla I*	720
EGFP-R	GCTTAATTAATCATTACACGGCGATCTTGCCG	*Pac I*	
ORFV-F1L-F	GCATCGATGCCACCATGGATCCACCCGAAATCAC	*Cla I*	1011
ORFV-F1L-R	GCTTAATTAATCATCACACGATGGCCGTGAC	*Pac I*	

Note: Underlined sequences indicate restriction sites.
