# Supplementary figures and images for: An ORFV F1L mRNA Vaccine Candidate: Preparation, Immunogenicity, and Comparison with a Commercial Live Vaccine
Source: Animals (Basel). 2026 Jul 22;16(14):2274. doi: 10.3390/ani16142274 (PMC13405235; doi:10.3390/ani16142274)

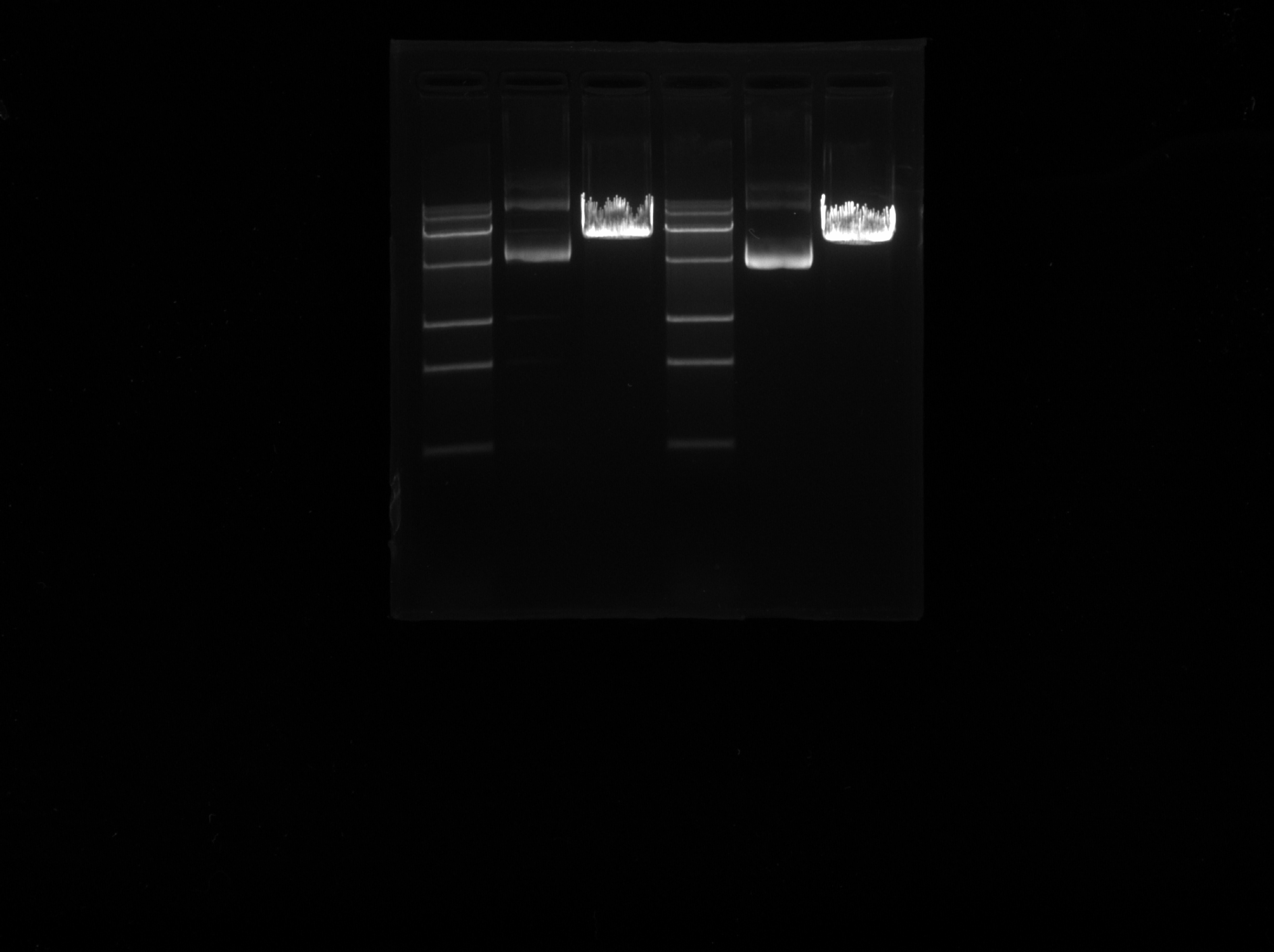

Supplement: Supplementary file 1 [file animals-16-02274-s001.zip › PCR and WB Raw Data/Fig .1 - (D) Double restriction enzyme digestion identification of recombinant plasmids pGEM-ORFV-F1L-UTR and pGEM-EGFP-UTR.jpg]

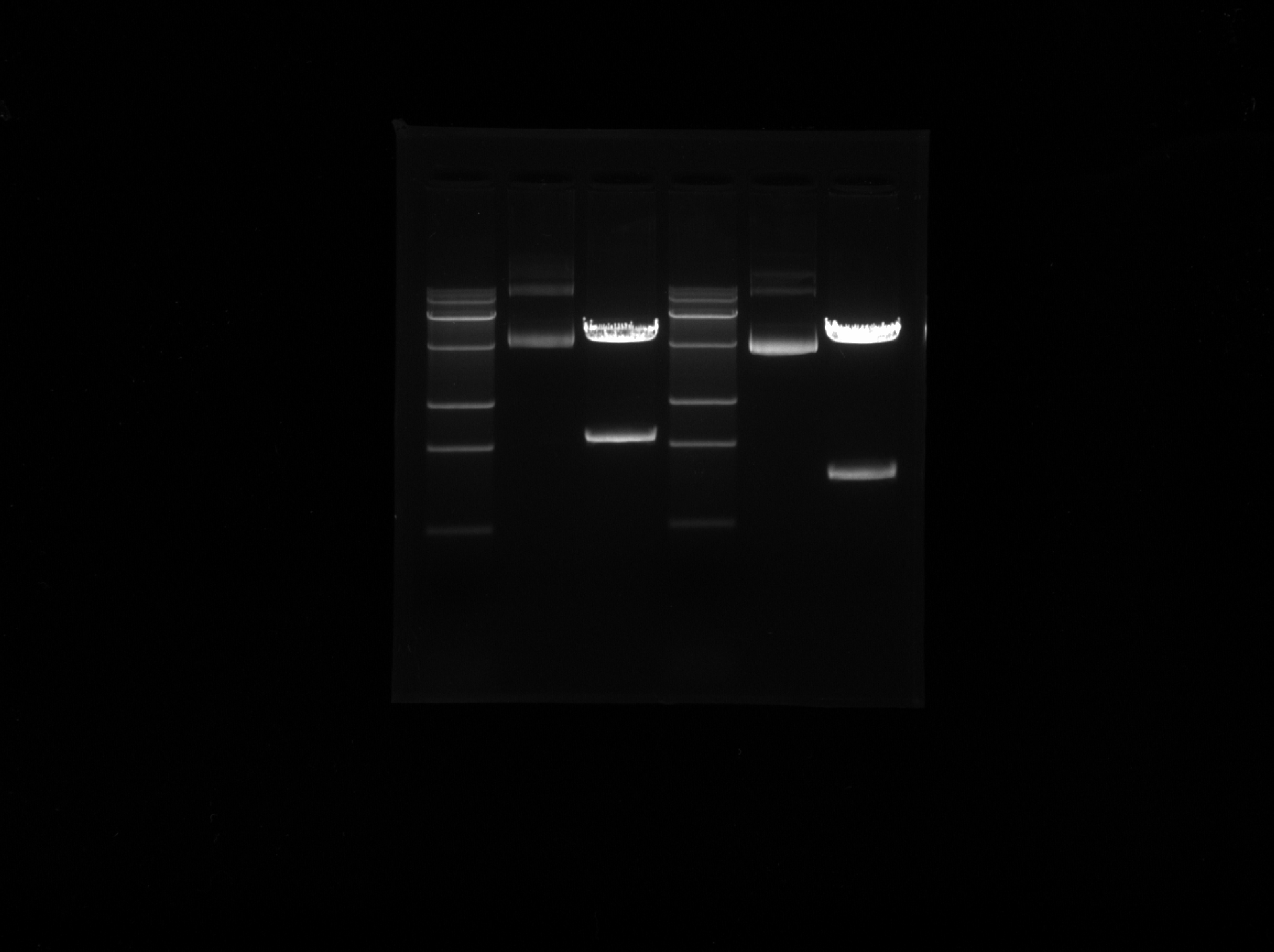

Supplement: Supplementary file 1 [file animals-16-02274-s001.zip › PCR and WB Raw Data/Fig. 1 -(C) Linearization identification of recombinant plasmids pGEM-ORFV-F1L-UTR and pGEM-EGFP-UTR by Xho I single restriction enzyme digestion..jpg]

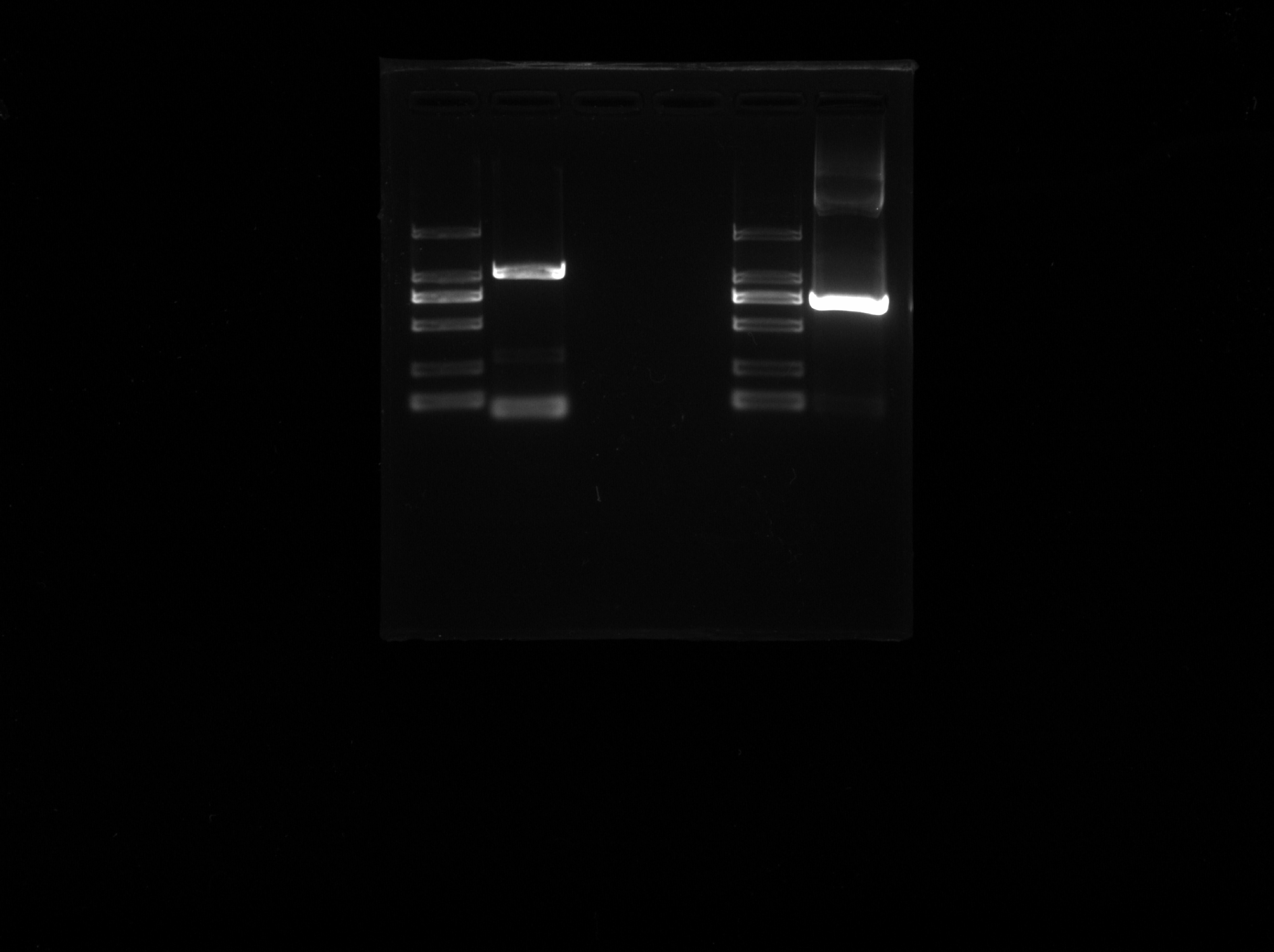

Supplement: Supplementary file 1 [file animals-16-02274-s001.zip › PCR and WB Raw Data/Fig.1-(A) (B) PCR amplification of ORFV-F1L and EGFP genes..jpg]

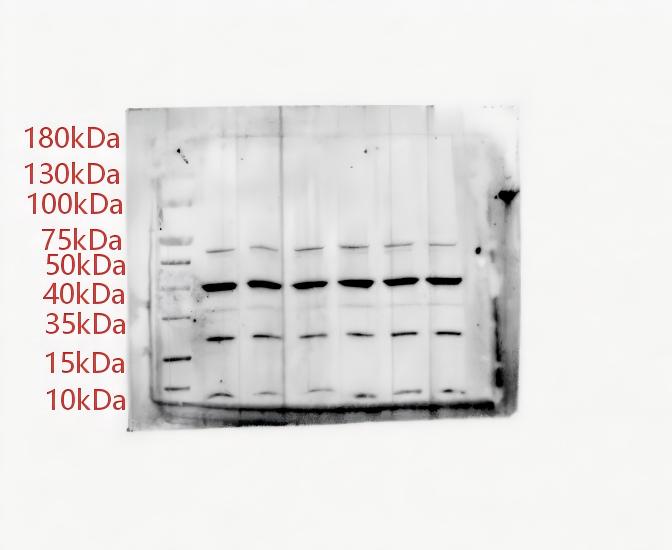

Supplement: Supplementary file 1 [file animals-16-02274-s001.zip › PCR and WB Raw Data/Fig.2 WB-ACTIN.jpg]

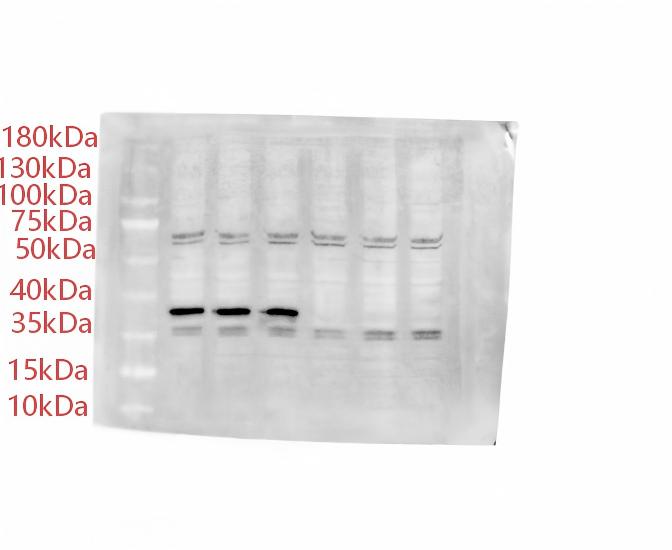

Supplement: Supplementary file 1 [file animals-16-02274-s001.zip › PCR and WB Raw Data/Fig.2 WB-F1L.jpg]
